# A case report of typical well-differentiated papillary mesothelial tumor diagnosed by internal thoracoscopy and literature review

**DOI:** 10.3389/fonc.2024.1359985

**Published:** 2024-04-09

**Authors:** Jian Guo, XiaoHui Lv, Wei Zhang, Fang Dong, Lei Li, Juan Liu, Bing Li

**Affiliations:** ^1^ Department of Respiratory and Critical Care Medicine, Shanghai Fourth People’s Hospital, Tongji University School of Medicine, Shanghai, China; ^2^ Department of Pathology, Shanghai Fourth People’s Hospital, Tongji University School of Medicine, Shanghai, China

**Keywords:** recurrent pleural effusions, well differentiated papillary mesothelial tumor, WDPMT, CA125, internal medical thoracoscopy

## Abstract

We report a case of well-differentiated papillary mesothelial tumor (WDPMT) diagnosed using internal thoracoscopic biopsy in a patient who has suffered from recurrent pleural effusions for over 35 years together with a history of elevated CA125. We hope to provide a case for the diagnosis of this rare benign and preinvasive pleural tumor and recommend that internal thoracoscopy may be a good choice in these recurrent pleural effusion patients especially for those minimal lesions not easily detected using CT scan.

## Brief medical history

**Figure 1:**
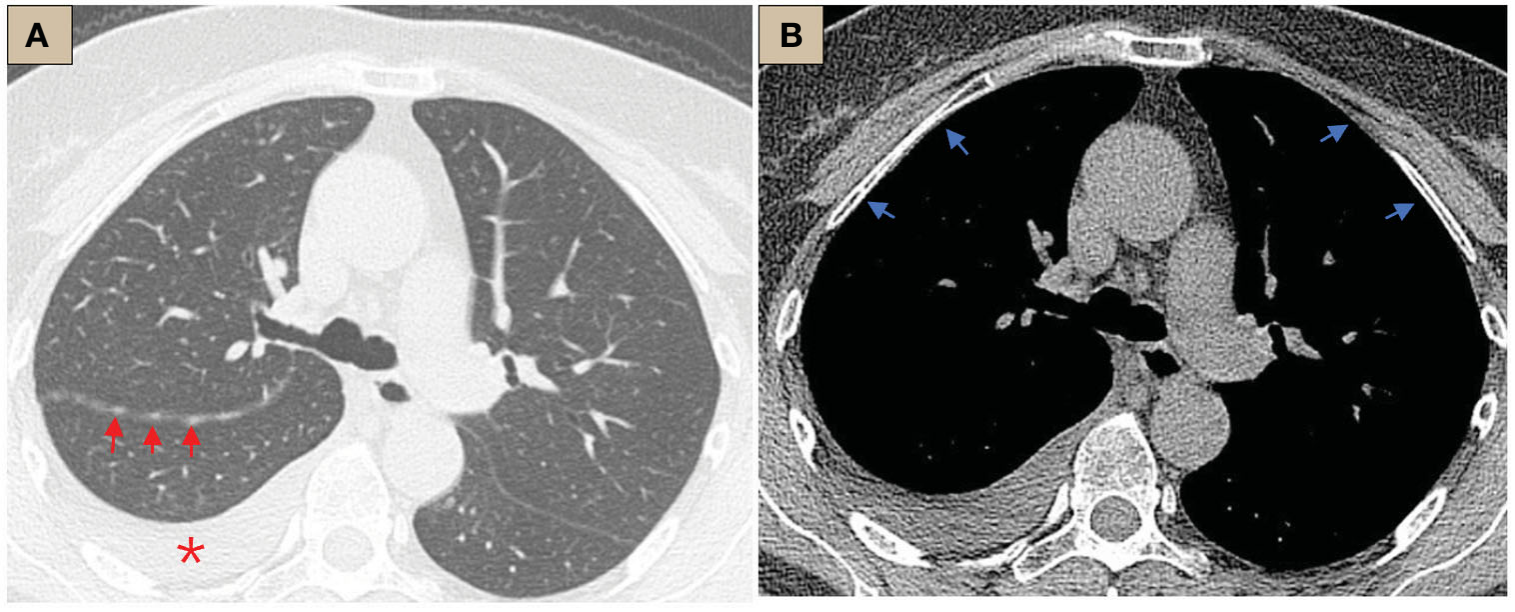
CT abnormalities before internal thoracoscopy examination. **(A)** CT lung window, tracheal bifurcation level, ×1-mm layer thickness, right pleural effusion (red *), right oblique fissure thickening, scattered nodules (red arrows); **(B)** CT mediastinal window, tracheal bifurcation level, ×1-mm layer thickness, pleura was smooth, no abnormal thickening or nodules (blue arrows).

The patient was a 64-year-old female who was admitted to the respiratory department because of cough and dyspnea for 1 week without fever, sputum, or other symptoms. CT scan suggested a small amount of right-sided pleural effusion and nodular thickening of the right oblique fissure, but the pleura in the mediastinal window was smooth (**Figure 1**). Careful inquiry of the past medical history revealed that this patient had recurrent right-sided pleural effusions over the past 35 years, which could date back to 1989. During the past 35 years, she had undergone at least four ultrasound-guided thoracentesis drains, but each puncture, whether by routine fluid test or pathologic or etiological test, did not reveal a definitive etiology. Another history was her CA125 level had been slightly elevated (approximately 130 U/ml, normal should be less than 35 U/ml) for over 20 years. There was no history of asbestos exposure. The patient is in good psychological condition and has no history of genetic disorders.

## Physical examination

Vital signs were as follows: body temperature 36.8°C, blood pressure 143/75 mmHg, heart rate 85 beats/min, respiratory rate 18 breaths/min, and oxygen saturation 98% (SpO_2_ measured by pulse oximeter). Respiratory rhythm was regular, no barrel chest or pestle finger. The lower border of the right lung was shifted upward along the scapular line. Auscultation of the right lung showed diminished breath sounds. Cardiac and abdominal examination showed no abnormal signs.

## Laboratory tests

Routine blood tests, white blood cell (WBC) 6.26 × 10^9^/L, hemoglobin (HGB) 126 g/L, neutrophilic granulocyte percentage 63.4%, blood platelet (PLT) 207 × 10_9_/L.

Arterial gas analysis, pH 7.45, partial pressure of carbon dioxide (PCO_2_) 4.49 kPa, Partial pressure of blood pressure (PO_2_) 16.2 kPa (2 L/min, nasal cannula oxygen inhalation).

Tumor markers, carcinoma embryonic antigen (CEA), alpha fetal protein (AFP), cancer antigen 153 (CA153), cancer antigen 199 (CA199), cancer antigen 724 (CA724), neuron-specific enolase (NSE) all in normal range. CA125 133.53 U/ml (normal range <35 U/ml).

CRP, ESR, interleukin-6 (IL-6), ADA, and autoantibodies tests were all in normal range.

Sputum smear tests twice for tuberculosis were negative twice.

Routine and biochemistry of pleural fluid, red blood cell (RBC): 1,000/µl, WBC 729/µl, lymphocytes: 87%, lactate dehydrogenase (LDH) 199.87 U/L, albumin (ALB) 52.11 g/L, glucose 3.8 mmol/L (glucose in blood 6.9 mmol/L).

*CRP, C-reactive protein; ESR, erythrocyte sedimentation rate; ADA, adenosine deaminase.

## Internal thoracoscopy

**Figure 2:**
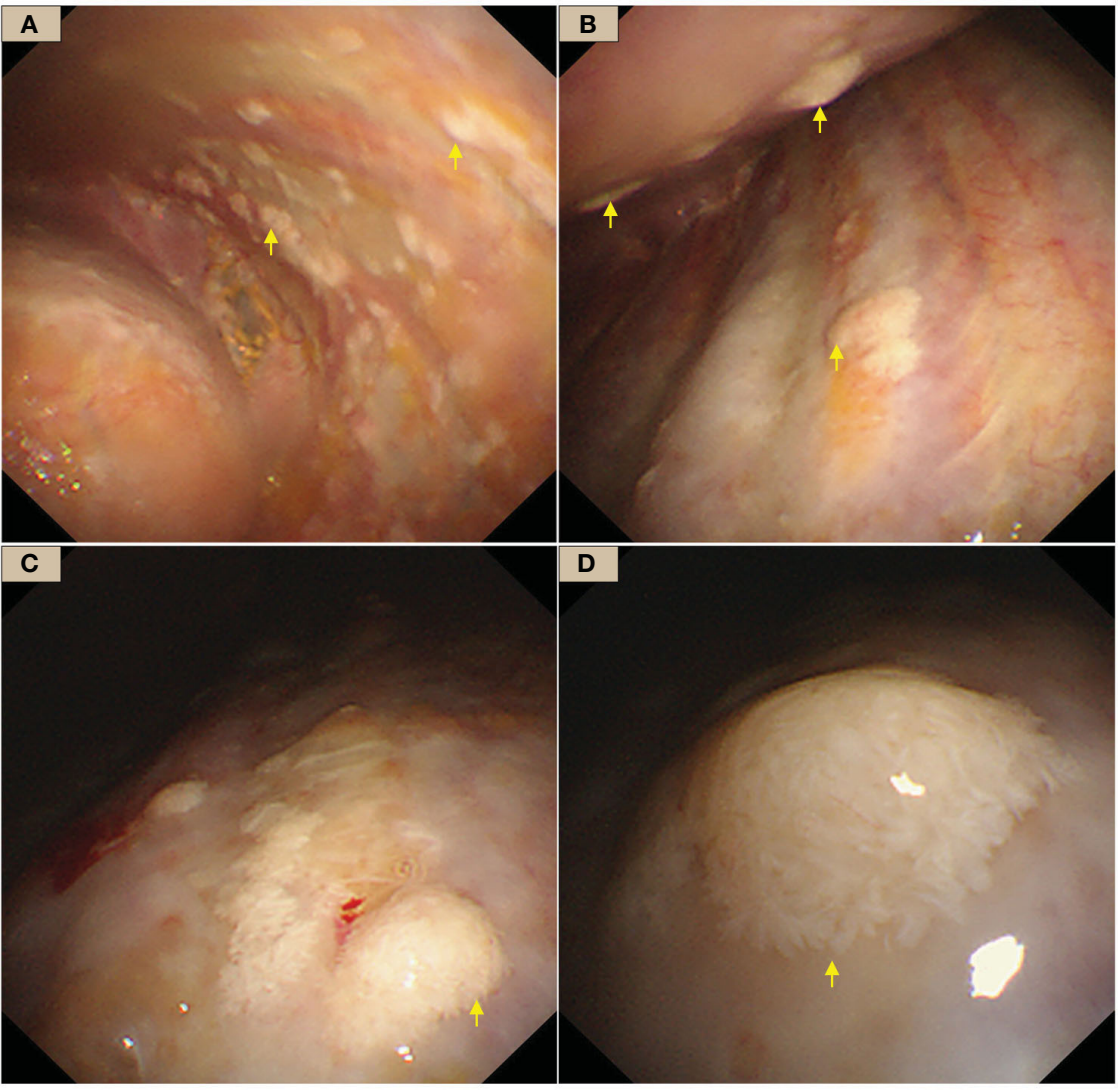
WDMPT presentations under internal thoracoscopy. **(A)** In the costal pleura near the diaphragm, scattered pale flattened lesions of various sizes (yellow arrows); **(B)** the anterior chest wall, at the level of the eighth rib, scattered nodules at both visceral and the parietal pleura with clear borders (yellow arrows); **(C)** mural pleural nodules of variable shapes and sizes (yellow arrows); **(D)** close view of the nodules, which are conical in shape and resemble a pile of feathers.

The patient had undergone several thoracentesis in other hospitals, but the etiology remains unclear, so we performed an internal thoracoscopy examination on 28/02/2023. The incision was made on the right midaxillary line at the seventh intercostal space. After bluntly separating the subcutaneous and muscular tissues in the pleural cavity, a trocar was inserted. Multifocal pale flat nodules were scattered over the pleura of the visceral and mural layers resembling a pile of snow or a pile of white feathers, or flattened white moss (**Figure 2**). The pleural cavity was filled with yellow clarified exudate. Ten intact lesions were biopsied with forceps (SpyBite Single-Use Biopsy Forceps, Radial Jaw 4) from different sites for pathologic examination. The nodules were soft and less likely bleeding.

## Pathologic report

**Figure 3:**
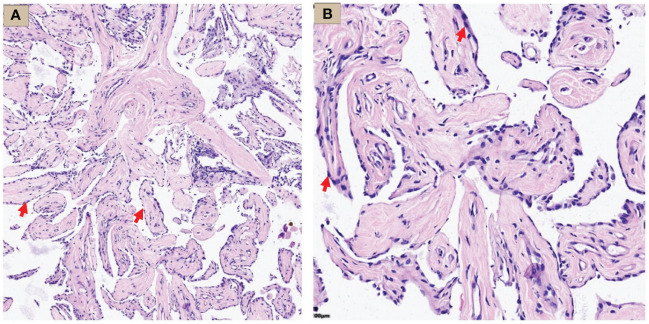
WDMPT HE staining results. **(A)** Low magnification: the tumor was composed of papillary structures (HE staining, ×100 magnification, red arrow); **(B)** high magnification: the papillary structure was covered by a monolayer of flat epithelial cells with rare infiltration of inflammatory cells (HE staining, ×200 magnification, red arrow).

Hematoxylin-Eosin (HE) staining showed that the tumor was composed of papillary structures covered with a single layer of mesothelial cells, flat or square, with rare nuclear divisions, no stromal or inflammatory infiltration. Most of the papillary stromal axis were composed of loose fibrous tissue, while fibroblasts and blood vessels were also seen in some areas (**Figure 3**).

**Figure 4:**
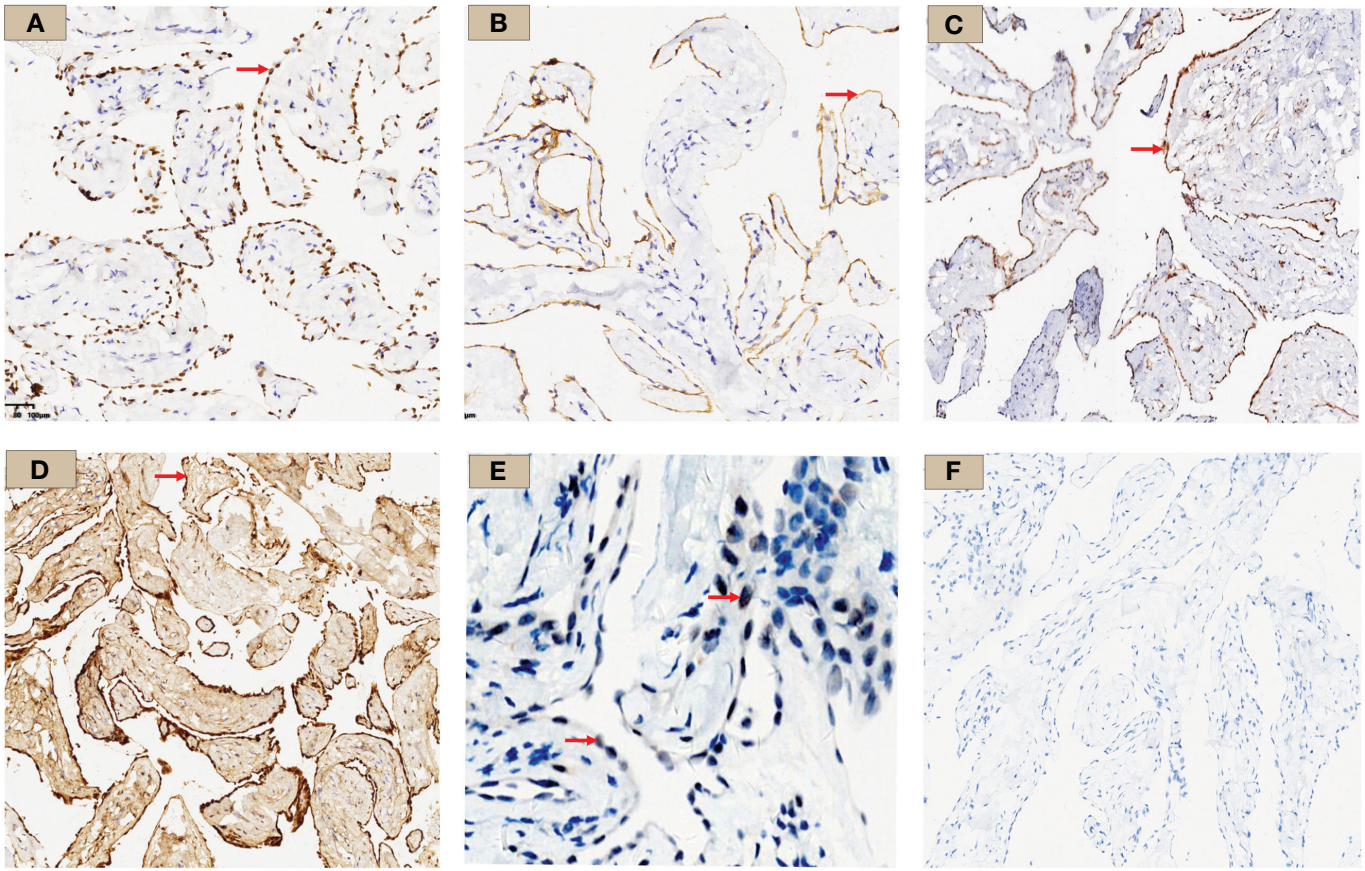
WT-1, D2-40, MTAP, Calretinin, BAP1, and PAX-8 immunohistochemistry results. **(A)** WT-1 staining: tumor cell nuclei was positively stained and shown in brown (×10, red arrow); **(B)** D2-40 staining: tumor cell membrane was stained brownish (×10, red arrow); **(C)** MTAP staining: tumor cell cytoplasm was stained brownish (×10, red arrow); **(D)** Calretinin staining: diffuse tumor cell cytoplasm and nucleus staining (×10, red arrow); **(E)** BAP1 staining: tumor cell nuclei stained; shown by a red arrow with red arrow (×10) **(F)** PAX-8 staining was negative in this case.

Immunohistochemistry showed strong positivity for WT-1, D2-40, and calretinin, suggesting the lesions originated from mesothelial cells. MTAP showed a diffuse positivity in tumor cell cytoplasm, and BAP1 was positive in the nuclei (**Figure 4**). Ki-67 proliferation index was approximately 2%. Other staining markers, including Ber-EP4, TTF-1, CDX-2, CK5/6, CK7, IMP3, P16, ER, PR, GATA-3, CD31, and ERG, were negative.

Based on histomorphology and IHC results, the diagnosis of well-differentiated papillary mesothelial tumor (WDPMT) was made.

## Discussion

Well-differentiated papillary mesothelial tumor (WDPMT), previously named well-differentiated papillary mesothelioma (WDPM), is a rare mesothelial benign and preinvasive tumor first reported by Yesner in 1953 (1, 2). However, these tumors are relatively indolent, histologically well differentiated, slow progressing, and most of the patients could have a long-term survival, which was significantly different from mesothelioma. In 2021, the WHO renamed WDPM to WDPMT to distinguish it from malignant mesothelioma (3).

Most literatures about WDPMT were case reports with approximately 200 cases since 1953. Abdominopelvic peritoneum in reproductive age female was the most common patient group and site of involvement. Nearly 90% in patients were discovered accidentally during other surgical procedures, and patients were asymptomatic. Different to malignant mesothelioma, the majority of patients do not have a clear history of asbestos exposure (4–6). In 2019, Guillaume Vogin reviewed 56 cases of WDPM from the RENAPE database occurring in the peritoneum, with a male-to-female ratio of 0.27, and a mean recurrence-free survival of 144 months during subsequent follow-up indicating female susceptibility and relatively better prognosis (7). In the same year, Meng Sun reported 75 cases of WDPM, which was the largest case series to date. In his report, 56% of the patients diagnosed with WDPM were in the age of 30–50 years, with a peak age of 40 years, and the male/female ratio was approximately 17/58. Thirty-one (41.3%) cases occurred in the peritoneum, followed by the greater omentum, mesentery, and fallopian tubes, and three cases were in the pleura (8). Different to peritoneum, Galateau-Sallé reported 24 cases of pleura WDPM without a difference in gender (male/female 11:13), but the age of onset was much later, and the majority of patients with pleural involvement had clinical symptoms such as dyspnea or recurrent pleural effusions (9, 10). The main clinical manifestation of our patient in this report was also recurrent pleural effusion, which could be traced back to 1989 at the earliest, but during these 35 years, the symptoms were generally mild, and no life-threatening or serious situation occurred, which was consistent with the benign clinical characteristics of WDPMT. In addition, this patient has a special history of elevated CA125; the first test in our hospital was 133.53 U/ml (reference range <35 U/ml), and by inquiring the history, the patient informed us she has a history of elevated CA125 for many years. During her treatment and follow up in our hospital, CA125 was totally checked five times ranging from 101.88 to 133.83 U/ml, and the most recent was on 25/12/2023, with a result of 133.83 U/ml. Due to the abnormally high CA125, we were very cautious in the diagnostic process because as far as we know, nearly no literature ever mentioned an elevated CA125 in WDPMT. To check the cause, especially gynecologic tumors, we performed HPV genotyping on cervical secretions, gynecological ultrasound, and pelvic MR, but no clear evidence of tumor was found. During follow-up, we closely monitored changes in CA125 and gynecologic ultrasound, although it has been reported in the literature that 1% of healthy women may have elevated CA125 (11, 12).

The most common imaging presentations of WDPMT are dendritic, single, or multiple nodules on viscera and parietal pleura, or scattered limited peritoneal thickening, while pleural and diaphragmatic calcifications are relatively rare, and the diameter of the nodules is often less than 1 cm (1, 5). In this case, CT scan did not show other abnormalities but right pleural effusion and oblique fissure thickening. However, under thoracoscopy, both visceral and parietal pleura were covered with various nodules, but these abnormalities were not observed in CT before we performed the operation, which suggest that the conventional imaging examination methods, such as CT and MR, may be challenging in detecting these lesions. Histologically, typical WDPMT is a papillary structure consisting of a single layer of epidermal cells without infiltration of inflammatory cells or stroma, and nuclear schizophrenic images are rare. For IHC, WDPMT also expresses WT-1, D2-40, and calretinin, but unlike malignant mesothelioma, BAP1 and MTAP were usually retained, whereas in malignant mesothelioma, these two markers are often diffusely absent. The 2021 WHO classification criteria also recommend BAP1 and MTAP or CDKN2A FISH as an important marker to differentiate WDPMT from malignant mesothelioma. Interestingly, Xing has reported that in their series, 61% of WDPMT patients could express PAX8, but we repeated PAX-8 staining three times, and the results were still negative. This may be related to the source of the WDPMT samples, as a significant proportion of the patients in Xing’s study were from gynecologic tumors, and PAX-8 is very common in normal cervical and endometrial epithelial cells as well as in gynecologic tumors (13, 14).

At the genetic level, some researchers have studied the mutations in WDPMT. Shrestha found that EHD1, ATM, FBXO10, SH2D2A, CDH5, MAGED1, and TP73 mutations are present in WDPMT, but they are rare in malignant mesotheliomas pointing out that these mutations may be specific to WDPMT (15). Another study suggesting the presence of TRAF7 or CDC42 missense mutations in WDPMT can be used to distinguish it from mesothelioma (16). However, in our case, no subsequent genetic testing was performed, and whether there was corresponding mutation remains unclear.

Regarding differential diagnosis, the patient’s inflammatory markers, including some tests to exclude tuberculosis infection, and biochemical routines of pleural effusion are not specific and are of limited suggestive values. The primary basis remains histopathology and immunohistochemical staining. Based on the patient’s history and the histopathologic results, we made the diagnosis of WDMPT.

In general, WDPMT is currently considered a benign or low-grade malignant tumor, and most patients may have a good prognosis, but recent studies have reported the transformation from WDPMT into mesothelioma during the follow-up (8, 17, 18). Churg explained this phenomenon that WDPMT may contain two components, true WDPTM and WDPTM *in situ* mesothelioma, which may be morphologically similar to WDPMT but can subsequently develop into a more malignant mesothelioma (9). This reminds us that the diagnosis of WDPTM needs to be made with extra caution. On one hand, sufficiently complete samples need to be obtained for pathologic examination. On the other hand, patients diagnosed with WDPMT should be closely followed up with regular review of chest imaging, including CT or ultrasound. In this case, after adequate drainage of pleural effusion and the diagnosis was made, we communicated with the patient, but she refused further chemotherapy or other treatments. We also believe that, although the patient has a long history of the disease, the overall condition tends to be mild. Finally, we made a multidisciplinary follow-up plan for every 6 months, including a regular review of CA125, pleural effusion ultrasound, and chest CT if necessary. The last follow-up was performed on 25/12/2023. The patient had no cough, no sputum, no shortness of breath, or no other discomforts. Only a small amount of pleural effusion was discovered through ultrasound. CA125 was 133.83 U/ml, similar to the previous tests. No intervention, but continuous follow up, was performed.

## Conclusions

We have summarized this case report to provide additional reference for the diagnosis of WDPMT. This patient reminds us to consider the possibility of this rare benign pleural tumor when patients present with recurrent pleural effusions. Second, in such patients, early internal thoracoscopy may be a good option as it is relatively less invasive and provides a direct vision of the chest cavity, particularly for those lesions not easily detected on images.

## Data availability statement

The raw data supporting the conclusions of this article will be made available by the authors, without undue reservation.

## Ethics statement

Written informed consent was obtained from the individual(s) for the publication of any potentially identifiable images or data included in this article.

## Author contributions

JG: Writing – original draft. XHL: Writing – original draft. WZ: Writing – review & editing, Methodology. FD: Writing – original draft. LL: Writing – original draft. JL: Writing – original draft. BL: Writing – review & editing.
